# Strategies for the Improvement of Pet Health and Welfare in Portugal Based on a Pilot Survey on Husbandry, Opinion, and Information Needs

**DOI:** 10.3390/ani10050848

**Published:** 2020-05-14

**Authors:** Joana Correia Prata

**Affiliations:** 1 Centre for Environmental and Marine Studies (CESAM) & Department of Chemistry, University of Aveiro, 3810-193 Aveiro, Portugal; 2 O Meu Animal, 4515-463 Porto, Portugal

**Keywords:** pet husbandry, pet diet, parasite control

## Abstract

**Simple Summary:**

This study examined husbandry practices in companion animals being conducted in Portugal in order to understand the health and welfare issues which can be improved. One of the more important findings is that, despite adopting most pets, pet owners do not adopt from animal shelters, possibly due to the large bureaucratic process. Veterinary practice is viewed as expensive and identification of animals with microchip is frequently disregarded. These issues may result in important threats to animal welfare and health and should be addressed at societal level. Several strategies based on results are proposed to improve pet health and welfare in Portugal, focusing on showcasing the importance of veterinary care, reducing the number of lost or abandoned animals, and improving awareness and education.

**Abstract:**

Pets are present in half of the homes across Portugal. However, little is known about the husbandry, opinion, and information needs of Portuguese pet owners. Thus, the objective of this work was to clarify this information providing the basis for suggesting potential improvements. Responses were collected through an online survey, including inhabitants from different regions of Portugal (*n* = 111). Cats and dogs are the most popular pets and the majority are adopted, fed commercial diets, live indoors, are vaccinated, dewormed, and treated for external parasites, and occasionally visit the veterinary practice. Portuguese owners are interested in improving their pet’s health, and would like to learn more about welfare, health assessment, and diet from veterinarians. However, microchip and municipal registration are often overlooked. Lack of adoption from animal shelters as well as expectations over the cost of veterinary practice were other difficulties identified in this study. Strategies for the improvement of pet health and welfare in Portugal were proposed as improving the perception of the value and importance of veterinary care, reducing the number of lost and abandoned pets, and improving awareness and education. Thus, Portuguese stakeholders and authorities must take the required measures to improve these issues.

## 1. Introduction

Portugal is a European country with a population of 10.5 million citizens [1], 1.4 million cats and 2 million dogs [2], with 54% of Portuguese homes having pets [3]. However, the country is still improving animal welfare such as the elevation of pets to sentient beings under legal protection [4] and the prohibition of euthanasia of healthy animals in overcrowded animal shelters [5]. Nonetheless, over 37,000 stray cats and dogs were collected from the streets by municipal entities in 2016 [6]. There is still a large gap between the perceptions and husbandry of pets in Portugal, with a perspective of mostly utilitarian use by rural communities facing the urban perspective as companion animals, sometimes to utopic extremes. The growing interest in the protection of companion animals in Portugal is represented by the growth of the political party PAN (Pessoas, Animais, Natureza; People, Animals, Nature), which has risen in votes from 1.4 to 3.3% in the past elections with a campaign mostly focused on pet welfare issues [7]. Indeed, companion animals worldwide are growing to be seen as family members [8], benefiting their owners by contributing to physical and psychological wellbeing, for instance, by improving social activities and daily exercise [9]. On the other hand, companion animals may contribute to exposure to zoonotic diseases, such as vector-borne diseases, if not properly managed [10].

Owner’s knowledge of preventive measures and husbandry are important factors in improving pet health and welfare. For instance, fewer behavioural problems and better welfare were reported for cats of owners with greater knowledge about their needs and behaviour [11]. Accidental litters of kittens, which may be relinquished to animal shelters, may also result from owners’ unfounded beliefs which could be reduced through educational interventions [12]. Veterinarians play an important role in the education of pet owners, which is also necessary for improving treatment compliance [13]. However, current owners’ husbandry practices and interests need to be assessed in order to create effective and engaging information and improve public awareness on animal rights and welfare. Stakeholders and legislators also require information to better understand problems involving the husbandry of pets. Indeed, data availability is essential for the identification and improvement of welfare and animal health threats in companion animals [14], especially when conducted at national level to better inform future policy decisions [15].

Despite being on the right track, Portugal still needs to improve animal welfare, such as access to veterinary care, compliance with pet identification laws, and reduction of animal abandonment and reproduction. However, both the general public and legislators lack information that may trigger effective changes in behaviour and politics. This information should cover Portuguese pet owners’ current practices as well as assess areas of interest that may acts as gateways to provide education. Therefore, the objective of this survey was to produce preliminary statistics on husbandry practices, owners’ opinions and self-reported information needs in Portugal, in order to identify major areas and strategies for the improvement of pet health and welfare and identify different husbandry practices between regions. In summary, these have been identified as the lack of adoptions from animal shelters, the perceived overpricing of veterinary care, and the lack of compliance with pet identification laws, while the major interests of Portuguese pet owners focused on welfare, health assessment, and diet. Some practices are regional, such as higher pet adoptions in Lisbon region. Hopefully, the major conclusions of this study can provide a starting ground for discussion and improvement of animal welfare in Portugal.

## 2. Materials and Methods

A survey, consisting on 21 questions composed of closed (multiple-choice, checkboxes) or short answers (e.g., participant age, number of animals in a household), was developed to obtain information on regions, animal groups, husbandry, opinion, and information needs of Portuguese pet owners. These questions included information about the owner (5), pets (11), owners’ opinion (4) and information needs (1) (Table A1). Most questions were comprised of multiple-choice options, while two were checkboxes where several options could be selected (type of pet, information needs), and four were short answers (owner’s name, owner’s email, owner’s age, and number of pets). Information about pets gathered data relative to factors that may have influence on health and welfare, questions which are often a part of a thorough anamnesis. Options on frequencies of preventive treatments for parasites and veterinary visits considered current recommendations (e.g., monthly, every three months, yearly). The opinion section mostly focused on questions being currently discussed on societal level, especially the costs of veterinary care and the role of private veterinarians in the treatment of stray animals, as well as perspectives on vaccination and gonadectomy procedures. Finally, the self-reported information needs of pet owners focused on nine topics which could be addressed independently on awareness and education campaigns. The estimated time for completion was 10 to 15 min. Personal information (owner’s name and email) were only collected to remove repeated entries and excluded from posterior data analysis. Portuguese regions were defined according to NUTS II: North, Center, Metropolitan Region of Lisbon (hereby Lisbon), Alentejo, Algarve, Autonomous Region of Azores (herby Azores) and Autonomous Region of Madeira (hereby Madeira). The survey was on created using Google Forms and distributed online on a pet information website (i.e., https://omeuanimal.com), social media (i.e., OMeuAnimal Facebook page and Portuguese speaking Facebook animal groups), and through an email campaign (i.e., OMeuAnimal email subscribers). The respondents were free to share the link for the survey on social media or through email. The survey was open from 12 of December 2017 to 4 of January 2018 and was valid only for responses collected from Portuguese residents above 18 years old. All respondents were informed and agreed to the collection of data for scientific purposes before answering the survey. Data was recorded in Excel 2016 and analyzed on SPSS version 24 using descriptive statistics and likelihood ratio, considering an α = 0.05. When using the likelihood ratio between regions, only regions > 5 responses were considered (North, Center, Lisbon regions).

## 3. Results

A total of 111 respondents answered the questionnaire, with 104 being females. The median age was 35.0 years, corresponding to 35.0 years in women and 36.0 years in men. Most of the respondents were located in the Lisbon (44.1%), North (27.0%) and Centre (19.8%) regions.

### 3.1. Portuguese Pet Population

Cats were the most frequent pet (80.2%), followed by dogs (56.7%), with 36.9% of the households having both cats and dogs. Interestingly, small mammals (e.g., rabbits, hedgehogs, hamsters) are more frequent in the Lisbon region, while the Centre region presents the highest percentage of birds, reptiles, turtles and fish (Table 1). Based on this sample, Portuguese pet owners seem to have a median of 2.0 pets. Since the Centre region reports a higher number of fish and birds, which are often held in numbers, it reports the highest number of animals per owner (4.0), however this value decreases to 3.0 when excluding fish owners.

### 3.2. Origin of New Pets

Regarding the origin, most animals were adopted (82.8%), directly collected as strays (36.9%), from other citizens (29.7%) and from animal rescues or shelters (16.2%). Adoption from citizens includes animals offered by friends as well as from strangers, mostly through the internet. Only 17.1% of animals were bought. The origin of the animals varies with the region, with adoption of strays directly from the street being the most frequent in the North and Lisbon regions, while adoption from citizens being more frequent in the Centre region (Table 2). However, this difference is not significant (LR_6_ = 11.612, *p* = 0.071).

### 3.3. Husbandry and Diet of Cats and Dogs

#### 3.3.1. Diet of Cats and Dogs

Regarding diet, most Portuguese owners prefer to feed their pets commercial diets (89.9%). However, 10.1% of owners do not use common commercial diets and prefer homemade diets or alternative diets (e.g., raw meat, vegetarian, grain free) (Table 3). Homemade diets are more frequent in the Centre (9.1%) and Lisbon regions (8.5%), while most alternative diets are fed in Lisbon (6.4%). However, differences in diets between regions are not significant (LR_6_ = 7.191, *p* = 0.304). In Portugal, when looking at differences between owners of dogs, cats, or both, the use of alternative diets were only reported when owning only cats (8.3%, *n* = 48), and the use of homemade diets were more frequent in owners of dogs (9.5%, *n* = 21) or owners of both cats and dogs (9.8%, *n* = 41).

#### 3.3.2. Environment

The majority of pets live indoors with no access to the exterior (71.8%), followed by indoors with controlled access to the exterior (16.4%). Only 7.3% of pets are reported to live permanently outside (Table 3). North and Centre regions have the highest numbers of animals living outdoors or living indoors with uncontrolled access to the outdoors. Living environment of pets are significantly different between the North, Centre and Lisbon regions (LR_8_ = 22.342, *p* = 0.004). Cats are most frequently kept permanently indoors (83.3%, *n* = 48) compared to dogs (66.7%, *n* = 21) or both cats and dogs (61.0%, *n* = 41).

#### 3.3.3. Preventive Treatment against Parasites

In this study, most animals were subjected to 2 to 4 preventive treatments for gastrointestinal parasites (59.5%) (Table 4), with no difference between regions (LR_6_ = 11.448, *p* = 0.075). In the North and Centre regions, where animals are most often kept outside (Table 3), this preventive treatment is done over 5 times a year in 13.3% and 9.1% of the cases, respectively. Nonetheless, a large portion of owners only conduct this treatment once a year (27.9%), which may be related to the predominance of pets living indoors. Preventive treatment for external parasites, such as fleas and ticks, is mostly done 2 to 4 times a year (52.3%), followed by a single treatment (18.0%) (Table 4), with significant differences between the North, Centre and Lisbon regions (LR_8_ = 23.286, *p* = 0.003).

#### 3.3.4. Vaccination and Veterinary Visits

Portuguese pet owners report to have vaccinated 91.9% of animals (Table 5), with no differences between regions (LR_2_ = 1.251, *p* = 0.535). Non-vaccinated animals live indoors, with 25 % having access to the outdoors. The general vaccination plans for pets in Portugal prevent highly contagious or potentially fatal diseases. For dogs, general vaccination includes rabies (obligatory), canine hepatitis, canine distemper, canine parvovirus, and leptospirosis, being dependent on risk of exposure vaccination against kennel cough and leishmaniasis. For cats, commonly administered vaccines protect against calicivirus, feline herpesvirus, and panleukopenia, being dependent on risk of exposure the administration of vaccines against feline immunodeficiency virus and feline leukaemia virus (FIV/FeLV). Most animals undergo 2 to 4 veterinary visits a year (57.7%), with 11.7% of the animals having more than 5 visits a year. There is no difference between regions regarding veterinary visits (LR_6_ = 3.464, *p* = 0.749).

#### 3.3.5. Microchip and Legal Registration

In Portugal, at the time of the survey (2017–2018), microchip was required by law for dogs, and all cats and dogs needed to be registered in the municipality services. Our results show that only 65.4% of pets had a microchip. Despite Lisbon having the lowest percentage (39.5%) of compliance (Table 5), no significant differences were found between regions (LR_2_ = 3.720, *p* = 0.156). However, this result includes cats, that were not required by law to be identified by microchip at the time of the survey. On the other hand, only 62.7% of pets were lawfully registered in the municipality, with Lisbon again having a low percentage of compliance (56.3%) (Table 5), a non-significant difference between regions (LR_2_ = 4.903, *p* = 0.086).

### 3.4. Opinion of Portuguese Pet Owners on Veterinary Practices

Pet owners were asked to express their opinion about important issues involving the practice of veterinary medicine in Portugal (Table 6). Veterinary medicine is regarded as expensive to owners (62.2%), possibly due to the lack of widespread pet health insurance that could help cover the costs of unexpected health problems. This perception is similar across regions (LR_2_ = 3.348, *p* = 0.188). On the other hand, 52.3% of pet owners believe veterinarians should treat stray animals brought by citizens and 17.1% believe this should be done free of charge. Again, this belief is shared equally across regions (LR_8_ = 11.227, *p* = 0.189). Most respondents report to be favourable to neutering and spaying pets (91.0%) and to vaccination (100%). Spaying or neutering pets is sometimes viewed as an unnecessary procedure that deprives the animal of a natural behaviour, sometimes even considered as a mutilation, reflecting in the 9.0% against it.

### 3.5. Information Needs of Portuguese Pet Owners

The final part of the questionnaire assessed the information needs of Portuguese pet owners, where they could choose more than one category that particularly interested them. The top three categories that owners would like to learn more about, especially from veterinarians, were welfare (47.7%), health assessment (45.0%), and diet (39.6%) (Table 7). These three categories express the focus on providing the best environment to preserve the pet’s health, as well as to quickly identify when the animal requires medical attention.

Relations between interest in learning about certain topics and husbandry practices were explored (Table A2 and Table A3). People interested in welfare, health assessment and diet do not report differences in husbandry regarding the origin of pets and frequency of veterinary visits. Additionally, interest in welfare and health assessment are not related with the pet’s living environment, while interest in diets is related to higher frequency of pets living indoors (LR_4_ = 11.975, *p* = 0.018). Similarly, owners concerned about welfare report higher compliance with vaccination protocols (98.1% vs. 86.2%, LR_1_ = 6.012, *p* = 0.014), likely recognizing the role of disease as a threat to welfare. While interest in diets does not translate into different feeding practices (LR_3_ = 2.327, *p* = 0.507), it relates to lower compliance with municipality registration (50.0% vs. 28.8%, LR_1_ = 5.057, *p* = 0.025) and higher support for spaying and neutering procedures (97.7% vs. 86.6%, LR_1_ = 4.797, *p* = 0.029). Other relationships found between interests and husbandry were learning about animal behaviour with a higher frequency of treating for internal parasites over 2 times a year (82.1% vs. 61.1%, LR_1_ = 7.429, *p* = 0.032), learning about vaccination with higher compliance with microchip application (78.6% vs. 57.4%, LR_1_ = 5.372, *p* =0.020), and learning about hygiene and higher use of homemade or alternative diets (13.6% vs. 9.2%, LR_3_ = 8.222, *p* = 0.042).

Since owners could select more than one topic of interest, some of these topics were highly related. Interest in vaccination was related to interest in breeds (83.3%, LR_1_ = 5.567, *p* = 0.018), hygiene (66.7%, LR_1_ = 10.572, *p* = 0.001), health assessment (58.0%, LR_1_ = 16.013, *p* < 0.001), diet (54.8%, LR_1_ = 6.434, *p* = 0.011), and common diseases (44.2%, LR_1_ = 8.186, *p* = 0.004). Interest in hygiene was related to interest in common diseases (70.8%, LR_1_ = 13.911, *p* < 0.001), welfare (66.7%, LR_1_ = 4.443, *p* = 0.035), behaviour (54.2%, LR_1_ = 4.693, *p* = 0.03), and breeds (20.8%, LR_1_ = 11.199, *p* < 0.001). Finally, interest in common diseases and health assessment were related (59.5%, LR_1_ = 5.741, *p* = 0.017). Positive opinion about neutering and spaying of animals was associated with the adoption of strays, from rescues and citizens (87.1% vs. 40.0%, LR_3_ = 15.863, *p* = 0.001), but the low number of responses against neutering and spaying should also be considered (*n* = 10). No other relationships were found regarding pet owners’ opinions and husbandry practices.

## 4. Discussion

A total of 111 respondents answered the survey, with most being females (*n* = 104), which may result from the nature of the dissemination medium (e.g., Facebook animal groups), the higher tendency for females to answer surveys [16], or their perceived role as primary caretakers of pets [17]. Similarly, the median age of 35 years for respondents was below the national median age of 44.8 years [1], probably due to the dissemination medium (i.e., online), typically used by a younger generation [18]. The North, Centre and Lisbon regions provided 91.0% of responses while only representing 84.0% of Portugal’s population [1]. The Lisbon region was slightly over-represented in the current study (44.1%) when compared to its residence population (27.4%). A limitation of this kind of study, that cannot be overcome, is that people who are more concerned about their pets are more likely to spend the time answering the survey, possibly causing a sampling bias. Despite identifying some regional patterns in pet care, these results should be merely indicative due to the limited number of respondents. Although limited by a small sampled population, these results are a good starting point for stakeholders and governments, which lacked data on pet owners’ practices up to this moment.

Cats (82.2%) were more frequent pets than dogs (56.7%). However, FEDIAF (European Pet Food Industry Federation) [2] reported a higher number of dogs than cats in Portugal. This is also in accordance with species visiting the Portuguese veterinarian practices in 2016, where dogs comprised 58.6% of business, followed by cats (38.6%) and other pet species (3.0%) [19]. Thus, it is likely that cats are overrepresented in this sample. Regarding the number of animals, the median of 2.0 pets was reported by pet owners answering the survey. In Portugal, the legal limits for the number of pets per household is 3 dogs, 4 cats, or 4 total pets for urban buildings [20]. For rural buildings, the number of pets allowed is 6, except when the area allows for a larger number of animals in good welfare conditions [20]. Indeed, the median of 2.0 animals per pet owner is within legal limits, with many homes owning both cats and dogs (36.9%). Most animals were adopted (82.8%), but only 16.2% from animal rescues and shelters. Purchased animals (17.1%) likely originated directly from breeders or licenced establishments, since commercialization of cats and dogs in pet shops has been largely limited in Portugal [21]. It is worth noting that, when answering the survey, owners could have considered pets that are usually not adopted, such as reptiles or birds. Moreover, breeding pets at home and other possibilities were not explored in this work. Most Portuguese owners feed commercial diets, with only 10.1% feeding alternative and homemade diets, which requires higher veterinary supervision. Similarly, 3.8% of pet owners in the USA fed their pets primarily on homemade diets and 12.7% on other alternative diets [22]. Most pets live indoors (71.8%), with significant differences between regions, likely due to the higher urbanization of the Lisbon region or higher number of cats, which mostly live permanently indoors (83.3%). Indeed, a similar survey conducted in Lisbon revealed that 90.0% of cats were exclusively kept indoors while 80.0% of dogs regularly had access to the outdoors [23]. This is also supported by another study in Lisbon that found that 50.0% of dogs visited the park daily [24]. Indeed, this study findings that 66.7% of dogs live exclusively indoors seem excessive considering the needs of the animals and likely results from an alternative interpretation of the survey or, instead, misconceptions regarding the importance of daily walks on the exposure of animals to the outdoors.

The treatment for endoparasites was conducted 2 to 4 times a year in 59.5% of cases. In a previous study conducted in Portugal, 61.3% of dogs and 69.1% of cats have been reported to suffer preventive treatments for intestinal parasites 3 to 4 times a year (Pereira et al., 2016), in agreement with these results. Indeed, prevalence of intestinal parasites in Portugal has been found to be 20.6% in Porto (North, [25]) and 33.0% in Lisbon [24], underlining the need for preventive measures. Similarly, treatment for external parasites is conducted 2 to 4 times a year in 52.3% of cases. In a previous study, ectoparasite treatment occurred monthly in 50.5% of dogs and 17.2% of cats [23]. The difference could result from the population sampled or social desirability bias caused by the face-to-face survey in the case of the cited work. For both internal and external parasites, the frequency of treatment should consider individual risk. Similarly, owners self-reported vaccination rates of 91.9%. Vaccination is an important procedure, especially for animals with access to the outdoors where they can be in contact with pathogenic agents [26]. Moreover, these preventive treatments may be related to the frequency of veterinary visits, namely of 2 to 4 a year (57.7%). This frequency is surprising considering that 62.2% of pet owners consider veterinary medicine as expensive, despite 52.3% expecting the involvement of private veterinarians in the treatment of strays. Frequent visits to the veterinary clinic could also be related to chronic diseases. However, the objective of the veterinary appointment was not evaluated in this questionnaire. This value is in accordance with the value reported for 2016 of 3.7 visits for dogs and 3.4 visits for cats [19]. Regarding legal matters, only 65.4% of pets had microchip and 62.7% municipality registration, which may contribute to the increasing numbers of lost or abandoned pets, since their owners cannot be easily identified. Nonetheless, it is worth noting that these observations were only based on a limited sample (*n* = 111), with low representation of some regions. Finally, owners identified welfare (47.7%), health assessment (45.0%), and diet (39.6%) as the main topics they would like to learn more about. Similarly, a survey in the USA identified the self-reported main topics searched online by pet owners as disease and treatment (e.g., treatment, procedures, alternatives) and health and prevention (e.g., diet, exercise, wellness, vaccinations) [27]. Thus, veterinarians should adapt preventive treatments and spend time informing owners about the best husbandry practices for each individual case.

Differences between regions were also considered since they could result from regional asymmetries and be useful in identifying priorities when planning interventions. Only the North, Centre, and Lisbon regions presented enough respondents to be compared, corresponding to 91.0% of the survey’s answers and to 84.0% of the Portuguese population [1]. No regional differences were found for diet type, compliance with vaccination protocols, number of veterinary visits, and microchip application. Marginal differences were found for the origin of pets, with Lisbon region having higher number of adoptions (89.8%), municipality registration, with Lisbon having the lowest compliance (56.3%), and with preventive treatment for internal parasites, with most Lisbon residents performing 2–4 treatments a year (73.5%). Although these differences are non-significant in the present study (0.05 < *p* < 0.09), this could result from study design and limited number of respondents, deserving further assessment in future studies. Significant differences between regions (*p* < 0.05) were found for the treatment of external parasites, with North reporting to perform more yearly treatments, and for pets’ living environments, with Lisbon having no pets living in the outdoors. Differences between regions could also result from social asymmetries in the access to wealth, health, and education [28]. These differences between regions should be considered when planning interventions.

The objective of this work was to identify major areas and strategies for the improvement of pet health and welfare in Portugal. Based on the previously described observations, three major areas of improvement were identified: (1) improving the perception of veterinary care and the importance of veterinary procedures; (2) reducing the number of lost or abandoned pets; and (3) improving awareness and education of pet owners. Therefore, interventions aiming to improve animal health and welfare in Portugal should be targeted at these areas. Suggestions on strategies to be implemented in Portugal are discussed in detail below.

### 4.1. Strategies for the Improvement of Pet Health and Welfare in Portugal

#### 4.1.1. Improving the Perception of Veterinary Care and the Importance of Veterinary Procedures

The first identified area of improvement is the need for better perception of veterinary medicine in Portugal. Veterinary medicine is a profession still lacking societal recognition, especially in its contribution to public health. For instance, in Portugal only 23% of veterinarians think they are well regarded by the general public [29]. Regardless of being able to produce positive health outcomes with limited resources to reduce costs, small animal practice is still perceived as expensive by 62.2% of Portuguese pet owners and 52.3% expecting the voluntary veterinary contribution in the treatment of stray animals, with 17.1% expecting it to be free. This expectation could result from the free access to national healthcare in humans practiced in Portugal, which befalls on veterinarians independently of their involvement in non-profit activities, such as low-cost spaying and neutering campaigns and collaborations with rescue groups. Conversely, 63.0% of Italians feel that the government should have responsibility over stray dogs and cats [30], despite not evaluating veterinary treatment of stray animals. In order to allow Portuguese private veterinarians to fill these expectations, funding is required which could be obtained by crowdfunding or greater governmental support.

Veterinary medicine is considered expensive by 62.2% of Portuguese pet owners. Similarly, a survey in the USA found that 62.0% of owners perceived veterinary services as very expensive, with 29% of owners admitting being unable to afford veterinary services [13]. Despite the perceived high cost, 57.7% of pets in Portugal visit the veterinary clinic or hospital 2 to 4 times a year, with 97.3% pets having veterinary care at least once a year, compared to only 70.0% in Italy [31]. Reasons for the lack of veterinary visits have been identified as the costs of veterinary care, inadequate understanding of the need for frequent prophylactic measures and clinical examination, and negative feelings about subjecting the animal to stress during transportation and examination [32]. Similarly, lack of pet health insurance is associated to disregarding the risk of disease, downplaying the value and complexity of veterinary care, considering euthanasia as an accessible option, and comparing expectations to human medicine [33].

Despite considering veterinary care expensive, 91.9% of Portuguese pets have up-to-date vaccination. This vaccination may include the obligatory rabies vaccine for dogs, which is an important public health measure to prevent this zoonosis [34], or other diseases, such as canine leishmaniasis, which has a prevalence of 19.2% in Lisbon [35]. Vaccination is especially important in the prevention of infectious diseases, which have been identified as the main cause of mortality in cats and dogs (63.7%) when analysing over 1000 pet deaths in France in 1999 [36]. Like veterinary visits, preventive treatments for internal and external parasites are mostly conducted 2 to 4 times a year (in 59.5% and 52.3% of pets, respectively). This frequency of treatment for endoparasites may be effective in some cases, such as in areas infested with *Toxocara* spp., while other cases may require monthly applications, such as in the case of animals exposed to *Echinococcus multilocularis* through hunting and eating small prey [37]. For ectoparasites, frequency will depend on individual exposure as well as duration and efficacy of the product being used [38]. Nearly half of pet owners admit frequently forgetting to re-administer preventive treatments for external and internal parasites (e.g., 42.0% in dogs), leading to an increased risk of infestation [23]. This is especially concerning when 50.0% Lisbon pet owners reported daily visits to public parks with their dogs, with 75.5% of animal being allowed to lick the owners’ faces, and 43.1% allowed to sleep in the owner’s bed, increasing the family’s risk of infestation [24].

The identified barriers and insufficiencies can be overcome with easy improvements, such as better communication and animal handling. Better communication can be achieved by improving veterinarian—client communication, through workshops and higher valorisation during veterinary education, and also clarifying the role of veterinary medicine in public health, through better representation in the media. Owners with stronger bonds with their pets visited the veterinarian 40% more often, which may also be translated in the overall frequency of veterinary practice visits, and better veterinary—client communication can improve the perceived value of the service [13]. The role in public health includes promotion of vaccination, which also improves pet’s health and longevity, as well adequate preventive treatment for parasites adapted to each case, by communicating the risk of parasitic zoonosis and setting automatic alarms for treatment re-administration (e.g., automatic emails or text messages). Animal stress during transportation and examination can be reduced by implementing low-stress handling procedures, with measures such as reducing luminosity, minimizing handling, using only subtle movements, covering carriers with towels, and reducing the presence of fearful animals in waiting rooms [39]. Finally, costs can be reduced through wider adoption of pet health insurance and better public financing for the treatment of stray animals. Insurance should be seen as an investment, allowing budgeting and protection in the case of unexpected disease while also improving animal health, as insured pets visit the veterinarian practice 30% more often [40]. However, insurance plans must be carefully assessed by pet owners, such as for including coverage for pre-existing diseases, and by veterinarians, in terms of compensation form and procedures covered [40]. Combination of these actions could result in better veterinary care for Portuguese pets by improving accessibility and recognizing its importance.

#### 4.1.2. Reducing the Number of Lost and Abandoned Pets in Portugal

The second identified area of improvement aims at reducing the number of lost and abandoned pets. Despite 82.8% of animals being adopted, reflecting the identification of animals as sentient being and owners as animal protectors [41], only 16.2% were adopted from shelters. This could be caused by the large bureaucracy involving the adoption process in animal rescue groups, including paperwork and house visits. Indeed, a survey conducted in the USA revealed that people avoided adopting pets from animal shelters due to their extensive requirements, which translated in lengthy adoption processes and often resulted in denials associated with negative emotions [41]. This is a serious problem since most Portuguese animal rescue groups or shelters are overcrowded. For instance, in 2016 municipal services reported having collected 37,077 and failing to collect 8339 animals, with almost all reporting being over-capacity [6]. Moreover, the current law prohibiting the euthanasia of healthy animals in shelters [5] was not accompanied with effective measures to prevent stray animals (e.g., widespread neutering campaigns), leading to an increasing number of sheltered animals and decreasing housing and welfare conditions. This law is also giving rise to large dog packs roaming both rural and urban areas, threatening the population, attacking farm animals, and creating a public health problem, with most municipalities lacking resources to capture and shelter these animals [42]. Suggestions that can lead to the reduction of the number of animals in shelters include: (a) reducing the requirements for pet adoption processes; (b) exposing the social issue of abandoned pets and the need for adoptions from animal shelters (e.g., increase public exposure to animal rescue groups) [41]; (c) creating better infrastructures, as distance and accessibility to animal shelters are major factors determining adoptions [43]; (d) creating “Temporary Adoption Programs” (also known as “Temporary Adoption Families”) where people can spend time with rescue animals without the obligation of adoption, which eventually leads to adoptions and reduced animal return rates [44]; (e) incentivizing the identification (e.g., microchipping) of pets in order to identify the owner of lost pets as well as increasing control measures; (f) legally protecting pets, such as requiring landlords to allow animals in the home; (g) improving the intervention of veterinarians in the prevention of behavioural problems which is a major cause of abandonment; and (h) incentivizing the spaying or neutering of animals by promoting it in veterinarian practices and creating low-cost widespread programs throughout the country [45].

The lack of compliance with electronic identification of pets also prevents the return to their families when lost or holding owners responsible for abandonment. In Portugal, only 65.4% of pets had microchip and 62.7% were registered in the municipality. Despite similar laws in most states in Australia, only 28% of dogs and 9% of cats entering shelters in Queensland in 2012 were identified as having a microchip [46]. Microchip application and registration of pets have been considered expensive by the pet owners, leading to lack of compliance [47,48]. Therefore, banning unnecessary bureaucracy, setting campaigns to provide free microchip applications by state veterinarians, and increasing inspections by the authorities may improve compliance.

At the time of the survey, difficulties were also caused by the existence of two concurrent databases for pet identification: (1) SICAFE (Sistema de Identificação de Caninos e Felinos, System of Canine and Feline Identification), optionally registered by the veterinarian at the time of microchip application and used for the recovery of lost pets; and (2) SIRA (Sistema de Identificação e Recuperação Animal, System of Animal Identification and Recovery), dependent on the compliance with municipality registration. These databases were merged in the end of 2019 to generate a single identification system, SIAC (Sistema de Informação de Animais de Companhia, Information System of Companion Animals), in which the animal is registered at the time of microchip application, providing a reliable database for pet identification and creating the opportunity for the posterior development of national pet health database, such as monitoring the legally required vaccination against rabies in dogs [49]. Implementation of the SIAC database, accompanied with the extension of obligatory microchip identification to cats and ferrets, besides dogs [50], will likely improve the recovery of lost animals, but only if compliance with identification is improved.

Finally, the number of unwanted litters must be reduced in order to prevent abandonment and improve adoption of already existing animals. In Portugal, only 9.0% of owners report to be against neutering or spaying procedures. In Ireland, owners identified barriers to gonadectomy as financial costs, perceived adequacy of existing controls besides neutering (e.g., keeping the pet indoors), and negative perceptions of the procedure (e.g., invasiveness, unnatural status, weight gain) [51]. Some of these are misconceptions, while other may be managed through information on better husbandry practices, such as reducing the risk of obesity caused by the increased feed intake and decreased activity after neutering [52] by controlled feeding of a low-caloric diet and fomenting daily exercise [53]. Information of positive impacts of gonadectomy may also improve perception about this procedure. Spaying and neutering pets avoids unwanted litters and reduces the risk of some diseases, such as mammary carcinoma in queens [54] and bitches [55], and prostatic hyperplasia in dogs [56]. Moreover, people who adopt animals are more supportive of neutering and spaying than those who buy, which could be related to a higher valorisation of purebred offspring, needing an increased awareness effort. Therefore, unwanted litters may be reduced by improving awareness of the positive effects of gonadectomy, creating social responsibility around pet ownership and reproduction, and offering campaigns of low-cost gonadectomies. Combined, the previously presented measures could reduce the number of animals looking for adoption and reduce the number of abandoned pets.

#### 4.1.3. Improving Awareness and Education of Portuguese Pet Owners

The third intervention, complementary to the previous solutions, is the need for increased education and awareness of Portuguese citizens on animal health, welfare, and husbandry. Pet owners are especially interested in welfare, health assessment, and diet, with vaccination and hygiene as the most frequently related to interest in other topics. Content targeted at these topics could act as a gateway to provide information in other needed areas, such as the need for microchip identification or following vaccination protocols. Education and awareness could be transmitted directly by the veterinarian, improving client loyalty, perception of quality of care, compliance with treatments and recommendations, and ultimately resulting in better pet care [13]. However, time limitations may make this impossible. Thus, on individual cases, veterinarians could recommend or share approved online content, while societal interventions may be required on general topics (e.g., need of neutering or spaying, need for preventive parasite treatment) using media and social media channels. These actions may be planned based on the previous assessment. For instance, the Lisbon region has the lowest number of purchased pets (10.2%) and therefore interventions targeted at owners of purchased purebred animals, such as the importance of gonadectomy procedures, would produce better results if targeted at other regions, such as the North and Centre regions.

Education could also benefit diet choices and practices, benefiting pet health. In Portugal, 10.1% of pets are fed homemade or alternative diets, which may pose a health risk, as these are often nutritionally inadequate and expose pets and family to dangerous microorganisms [57]. For instance, both commercial and homemade raw foods often present contamination with *Escherichia coli* and *Salmonella enterica* [58], are usually nutritionally imbalanced (e.g., deficient in calcium, excessive in vitamin D) [59], and may increase plasma thyroxine causing dietary hyperthyroidism in dogs [60]. Similarly, homemade diets are often nutritionally imbalanced due to deficient owner information, difficulties in following strict recipes, and variations in ingredient quality [57]. In cats, vegetarian diets are known to be deficient in an essential amino acid (taurine), potentially causing dilated cardiomyopathy, and to produce a more alkaline urine, predisposing to the crystallization of urine salts and urolithiasis [61]. While any diet can be nutritionally adequate for pets if balanced and complete, independently of ingredients, alternative diets tend to be unbalanced more often or present microbiological contamination. Thus, Portuguese veterinarians may need to improve monitoring of pets, with more regular check-ups, and provided guidance for pet owners feeding these diets.

Despite finding that most pets live indoors, there is still 7.3% which live permanently outside. While 83.3% of cats live exclusively indoors, this is only true for 66.7% of dogs, with previous studies in Portugal suggesting that 80.0% of dogs had regular access to the outdoors [23]. Access to the outdoors may increase exposure to pathogens and parasites. For instance, outdoor access in cats is related to higher prevalence of infection and parasitism compared to indoor environments, including of zoonotic agents such as *Toxoplasma gondii* [62]. Similarly, dogs housed outdoors are more likely to have parasites, such as *Toxocara* spp. [63]. Therefore, there is a need for strict preventive treatments and regular veterinary check-ups to preserve the pet’s and their family’s health. Moreover, self-perceived information needs may not represent the needs for public health. For instance, lack of knowledge about transmission routes of internal parasites, lack of understanding of zoonotic risks to human health, and lack of preventive treatment for internal parasites in human beings, especially in pet owners, are public health concerns that shall be addressed through education despite not being identified in the present study [23]. Indeed, owner education is one of the major factors determining pet health and welfare, and possibility the proposed intervention with the highest cost return.

## 5. Conclusions

An online survey was conducted in order to collect information on husbandry, opinion and information needs of Portuguese owners and identify strategies for the improvement of animal health and welfare in Portugal. Most Portuguese pets are adopted, eat commercial diets, live indoors, are frequently treated for intestinal and external parasites (2–4 times a year), are vaccinated and occasionally visit the veterinary practice (2–4 times a year). Portuguese owners revealed to be extremely worried about their pet’s health and seek further information to meet this goal. However, legal matters such as microchip and municipal registration are still overlooked by Portuguese owners, requiring further sensitization and enforcement. This lack of identification, the growing number of abandoned pets, and the unpopularity of adopting animals from shelters contribute to the increasing numbers of lost or abandoned animals looking for families, requiring intervention by the authorities and stakeholders. Dissonance between the expected and real costs of veterinary practice in Portugal may be overcome through better communication between veterinarians and pet owners, as well as through pet insurance plans that provide better access to animal health, and crowd funding or increased government support in the case of stray animals. Therefore, strategies for the improvement of pet health and welfare in Portugal must focus on improving the perception of veterinary care and the importance of veterinary procedures, reducing the number of lost and abandoned pets, and improving awareness and education of pet owners. Despite the identified trends, it is worth mentioning that the present study only presents an initial assessment of the Portuguese situation regarding pet care, requiring more research to better clarify and update this data.

## Figures and Tables

**Table 1:** Portuguese pet owners (%) reporting to have different animal types in their household, by region.

Region	*n*	Dogs	Cats	Small Mammals	Birds	Reptiles	Turtles	Fish
North	30	56.7	76.7	3.3	13.3	0.0	10.0	20.0
Centre	22	68.2	86.4	0.0	18.2	4.5	18.2	27.3
Lisbon	49	51.0	79.6	10.2	10.2	0.0	8.2	8.2
Alentejo	3	33.3	100	0.0	0.0	0.0	33.3	0.0
Algarve	2	100	0.0	0.0	50.0	0.0	50.0	0.0
Azores	4	50.0	100	0.0	0.0	0.0	25.0	0.0
Madeira	1	0.0	100	0.0	0.0	0.0	0.0	0.0
Total	111	55.9	80.2	5.4	12.6	0.9	12.6	14.4

**Table 2:** Origin (%) of the pets owned in Portugal.

Region	*n*	Purchased	Adopted from Shelters	Collected as Stray	Adopted from Citizens
North	30	26.7	16.7	43.3	13.3
Centre	22	22.7	13.6	18.2	45.5
Lisbon	49	10.2	16.3	40.8	32.7
Alentejo	3	0.0	33.3	33.3	33.3
Algarve	2	0.0	0.0	100	0.0
Azores	4	25.0	25.0	25.0	25.0
Madeira	1	0.0	0.0	0.0	100
Total	111	17.1	16.2	36.9	29.7

**Table 3:** Husbandry of Portuguese pets: diet and living environment.

Region	*n*	Diet (%)			Environment (%)			
		CM	HO	AL	IN	IC	IUC	OU
North	30	96.7	3.3	0.0	63.3	20.0	10.0	6.7
Centre	22	86.4	9.1	1.0	59.1	9.1	9.1	22.7
Lisbon	49	85.1	8.5	6.4	83.3	16.7	0.0	0.0
Alentejo	3	100	0.0	0.0	66.7	33.3	0.0	0.0
Algarve	2	100	0.0	0.0	100.0	0.0	0.0	0.0
Azores	4	100	0.0	0.0	50.0	25.0	0.0	25.0
Madeira	1	100	0.0	0.0	100	0.0	0.0	0.0
Total	111	89.9	6.4	3.7	71.8	16.4	4.5	7.3

Diet: CM—commercial diet; HO—homemade diet; AL—alternative diet. Environment: IN—indoors; IC—indoors with controlled access to outdoors; IUC—indoors with uncontrolled access to outdoors; OU—outdoors.

**Table 4:** Husbandry of Portuguese number of yearly preventive treatment for internal and external parasites.

Region	*n*	Internal Parasites (%)				External Parasites (%)				
		0	1	2–4	>5	0	1	2–4	5–10	>10
North	30	0.0	40.0	46.7	13.3	4.5	30.0	30.0	30.0	10.0
Centre	22	9.1	31.8	50.0	9.1	8.2	18.2	72.7	0.0	4.5
Lisbon	49	4.1	16.3	73.5	6.1	0.0	10.2	53.1	18.4	10.2
Alentejo	3	0.0	33.3	66.7	0.0	0.0	33.3	33.3	0.0	33.3
Algarve	2	0.0	50.0	0.0	50.0	0.0	0.0	100	0.0	0.0
Azores	4	0.0	50.0	50.0	0.0	0.0	25.0	75.0	0.0	0.0
Madeira	1	0.0	0.0	100	0.0	0.0	0.0	100	0.0	0.0
Total	111	3.6	27.9	59.5	9.0	4.5	18.0	52.3	16.2	9.0

**Table 5:** Husbandry of Portuguese pets: vaccination status (%), identification with microchip (%), municipality registration (%), and veterinary visits per year (%).

Region	*n*	Vaccine		Microchip		Registered		Veterinary Visits per Year			
		Yes	No	Yes	No	Yes	No	0	1	2–4	>5
North	30	90.0	10.0	73.3	26.7	70.0	30.0	3.3	26.7	56.7	13.3
Centre	22	90.9	9.1	81.8	18.2	81.8	18.2	0.0	31.8	63.6	4.3
Lisbon	49	95.9	4.1	39.5	60.4	56.3	43.8	2.0	26.5	55.1	16.3
Alentejo	3	100	0.0	66.7	33.3	66.7	33.3	0.0	66.7	33.3	0.0
Algarve	2	100	0.0	50.0	50.0	50.0	50.0	0.0	50.0	50.0	0.0
Azores	4	50.0	50.0	0.0	100	0.0	100	25.0	0.0	75.0	0.0
Madeira	1	100	0.0	0.0	100	0.0	100	0.0	100	0.0	0.0
Total	111	91.9	8.1	65.4	34.5	62.7	37.3	2.7	27.9	57.7	11.7

**Table 6:** Opinion of Portuguese pet owners on veterinary practice prices (%), treatment of stray animals (%), spaying and neutering (%), and vaccination of pets (%).

Region	*n*	Prices			Treatment of Strays					Spaying		Vaccination	
		C	A	E	MR	NA	NC	OP	ON	Yes	No	Yes	No
North	30	0.0	50.0	50.0	16.7	3.3	63.3	10.0	6.7	86.7	13.3	100	0.0
Centre	22	0.0	45.5	54.5	13.6	9.1	50.0	4.5	22.7	86.4	13.6	100	0.0
Lisbon	49	0.0	30.6	69.4	4.1	8.2	49.0	18.4	20.4	93.9	6.1	100	0.0
Alentejo	3	0.0	33.3	66.7	33.3	0.0	33.3	0.0	33.3	100	0.0	100	0.0
Algarve	2	0.0	0.0	100	0.0	0.0	50.0	0.0	50.0	100	0.0	100	0.0
Azores	4	0.0	0.0	100	0.0	25.0	25.0	50.0	0.0	100	0.0	100	0.0
Madeira	1	0.0	100	0.0	0.0	0.0	100	0.0	0.0	100	0.0	100	0.0
Total	111	0.0	37.8	62.2	9.9	7.2	52.3	13.5	17.1	91.0	9.0	100	0.0

Prices of veterinary care: C—cheap; A—average; E—expensive; Treatment of strays: MR—may refuse, NA—not obliged, but should treat animals brought by animal rescue groups, NC—not obliged, but should treat animals brought by citizens, OP—obliged, with payment from citizen, ON—obliged, without payment from citizen.

**Table 7:** Portuguese pet owners (%) reporting being interested in learning new information on a topic.

Region	*n*	D	W	B	T	V	H	C	H	B
North	30	46.7	43.3	33.3	13.3	33.3	30.0	26.7	16.7	3.3
Centre	22	18.2	45.5	22.7	4.5	45.5	45.5	36.4	13.6	4.5
Lisbon	49	46.9	57.1	40.8	12.2	36.7	55.1	40.8	24.5	8.2
Alentejo	3	66.7	0.0	33.3	33.3	66.7	33.3	0.0	33.3	0.0
Algarve	2	50.0	100	50.0	50.0	100	100	100	100	0.0
Azores	4	0.0	0.0	25.0	0.0	0.0	25.0	100	25.0	0.0
Madeira	1	0.0	0.0	100	100	0.0	0.0	0.0	0.0	0.0
Total	111	39.6	47.7	35.1	12.6	37.7	45.0	37.8	21.6	5.4

D—diet; W—welfare; B—behaviour; T—training; V—vaccination; H—health assessment; C—common diseases; H—hygiene; B—breeds.

## Appendix A

**Table A1:** Questionnaire used to assess Portuguese pet owners’ husbandry practices, opinions and self-reported information needs.

Section	Question	Options	Type of Question
Owner’s information	Name	n.a.	Short answer
	Email	n.a.	Short answer
	Age	n.a.	Short answer
	Sex	Male/female/other	Multiple option
	Region	North/Center/Lisbon metropolitan region/Alentejo/Algarve/Autonomous region of Azores/Autonomous region of Madeira	Multiple option
Pet information	Type of pets owned	Dog/cat/turtle/fish/bird/small mammals/others	Checkboxes
	Number of owned animals	n.a.	Short answer
	Origin of the pets	Bought/adopted from a shelter/adopted from other citizens/adopted from the street	Multiple option
	Diet type of cat and dog	Commercial/homemade/alternative (grain free, no carbohydrate, vegetarian, raw)	Multiple option
	Living environment of cat or dog	Indoors (inside)/outdoors (outside)/indoors with uncontrolled access to the outdoors/indoors with controlled access to the outdoors	Multiple option
	How many times a year do you preventively treat pets for internal parasites (e.g., tenias, worms)?	None/1/2–4/>5	Multiple option
	How many times a year do you preventively treat pets for external parasites (e.g., fleas, ticks)?	None/1/2–4/5–10/>10	Multiple option
	Do you vaccinate your pets as recommended by your veterinarian?	Yes/no	Multiple option
	Does your cat or dog have microchip identification?	Yes/no	Multiple option
	Does your cat or dog have up-to-date municipality registration?	Yes/no	Multiple option
	How many times does your pet visit a veterinary establishment in a year?	None/1/2–4/>5	Multiple option
Owners’ opinions	What is your opinion about the price of veterinary care?	Cheap/not cheap nor expensive/expensive	Multiple option
	What is your opinion on the treatment of stray animals by veterinarians?	They are obliged to treat without costs to citizens/they are obliged to treat sharing the costs with citizens/they are not obliged but should treat strays brought by citizens/they are not obliged but should treat strays brought by shelters and rescues/they can refuse	Multiple option
	What is your opinion about spaying and neutering pets?	I am in favor/I am against	Multiple option
	What is your opinion about the vaccination of pets?	I am in favor/I am against	Multiple option
Information needs	What are the main questions or topics which you would like your veterinarian to address?	Diet/welfare/behavior/training/vaccination/health assessment/most common diseases/hygiene/breeds	Checkboxes

n.a. not available.

**Table A2:** Likelihood ratios between the interests of owners and other variables.

Variable	Breeds	Behavior	Training	Vaccination	Common Diseases	Hygiene	Diet	Welfare	Health
Origin	0.656	0.492	0.623	0.197	0.327	0.144	0.385	0.617	0.401
Deworming (Int)	0.091	0.032	0.676	0.453	0.190	0.199	0.617	0.062	0.580
Deworming (Ext)	0.298	0.188	0.414	0.497	0.638	0.405	0.850	0.899	0.275
# Veterinary visits	0.481	0.362	0.413	0.198	0.395	0.529	0.158	0.181	0.806
Diet type	0.191	0.538	0.410	0.511	0.889	0.042	0.507	0.722	0.065
Environment	0.794	0.075	0.592	0.379	0.397	0.272	0.018	0.181	0.730
Vaccination	0.307	0.381	0.111	0.296	0.770	0.964	0.684	0.014	0.970
Microchip	0.316	0.825	0.253	0.020	0.306	0.639	0.253	0.082	0.608
Registration	0.836	0.312	0.646	0.278	0.176	0.836	0.025	0.276	0.886
Veterinary care prices	0.534	0.359	0.681	0.965	0.718	0.969	0.348	0.679	0.121
Veterinary care of strays	0.165	0.506	0.133	0.254	0.295	0.708	0.449	0.632	0.183
Spaying or neutering	0.540	0.056	0.092	0.883	0.203	0.311	0.029	0.606	0.083
Vaccination	n.a.	n.a.	n.a.	n.a.	n.a.	n.a.	n.a.	n.a.	n.a.

n.a. not available. # number.

**Table A3:** Likelihood ratios between opinions of owners and other variables.

Variable	Veterinary Care Prices	Veterinary Care of Strays	Spaying or Neutering	Vaccination
Origin	0.249	0.136	0.001	n.a.
Deworming (Int)	0.865	0.545	0.693	n.a.
Deworming (Ext)	0.456	0.798	0.424	n.a.
# veterinary visits	0.924	0.294	0.708	n.a.
Diet type	0.065	0.240	0.332	n.a.
Environment	0.084	0.475	0.737	n.a.
Vaccination	0.134	0.126	0.525	n.a.
Microchip	0.673	0.599	0.823	n.a.
Registration	0.729	0.602	0.290	n.a.

n.a. not available. # number.
